# Dashboard-Guided Anti-TNF Induction: An Effective Strategy to Minimize Immunogenicity While Avoiding Immunomodulators—A Single-Center Cohort Study

**DOI:** 10.1093/crocol/otaf023

**Published:** 2025-06-27

**Authors:** Elena Céspedes-Martínez, Virginia Robles-Alonso, Xavier Serra-Ruiz, Claudia Herrera-De Guise, Luis Mayorga-Ayala, Sonia García-García, María Larrosa-García, Francesc Casellas, Natalia Borruel

**Affiliations:** Unitat d’Atenció Crohn Colitis (UACC), Gastroenterology Department, Vall d’Hebron University Hospital, Barcelona, Spain; Unitat d’Atenció Crohn Colitis (UACC), Gastroenterology Department, Vall d’Hebron University Hospital, Barcelona, Spain; Unitat d’Atenció Crohn Colitis (UACC), Gastroenterology Department, Vall d’Hebron University Hospital, Barcelona, Spain; Unitat d’Atenció Crohn Colitis (UACC), Gastroenterology Department, Vall d’Hebron University Hospital, Barcelona, Spain; Unitat d’Atenció Crohn Colitis (UACC), Gastroenterology Department, Vall d’Hebron University Hospital, Barcelona, Spain; Pharmacy Department, Vall d’Hebron University Hospital, Barcelona, Spain; Pharmacy Department, Vall d’Hebron University Hospital, Barcelona, Spain; Unitat d’Atenció Crohn Colitis (UACC), Gastroenterology Department, Vall d’Hebron University Hospital, Barcelona, Spain; Unitat d’Atenció Crohn Colitis (UACC), Gastroenterology Department, Vall d’Hebron University Hospital, CIBER-EHD, Barcelona, Spain

**Keywords:** tumor necrosis factor inhibitors, pharmacokinetics, decision support systems, immunogenicity, therapeutic drug monitoring

## Abstract

**Background:**

Proactive therapeutic drug monitoring facilitates early dose optimization to prevent primary and secondary failure to antitumor necrosis factor (TNF). We aimed to investigate the impact of dashboard-guided induction dosing strategy on anti-TNF durability and immunogenicity.

**Methods:**

We conducted a single-center cohort analysis of patients with Crohn’s disease (CD) and Ulcerative colitis (UC) who initiated treatment with infliximab or adalimumab between January 2020 and March 2023. Induction was prospectively personalized using a pharmacokinetic model-guided dosing strategy, with drug measurements at week 2, 6, and 14, and the first dose adjustment occurred in week 4. Data were recorded retrospectively. We assessed treatment durability, pharmacokinetic outcomes, clinical remission (CR), and endoscopic remission (ER), at both weeks 24 and 56. Multivariate analysis and Kaplan–Meier curves were used to compare outcomes.

**Results:**

We enrolled 147 patients (92 CD /55 UC). Anti-TNF drug survival probability was 85.00% after a year. Seventy-seven percent of patients were prescribed an intensified dose in the first year, which was associated with improved drug durability. Only 1 patient out of 147 developed antibodies to adalimumab, none to infliximab. After 24 and 52 weeks of treatment 92.5% (136/147) and 72.78% (107/147) of patients achieved CR, respectively. ER was observed in 59.39% (79/133) of patients. The use of immunomodulators or carriage of HLA DQA1*05 variant was not associated with adverse treatment or pharmacokinetic outcomes.

**Conclusions:**

Optimizing anti-TNF induction with a dashboard-guide dosing strategy proves to be a valuable approach to enhance treatment durability and clinical outcomes in inflammatory bowel disease patients. Immunogenicity appears to be mitigated by the model, which even mitigates the impact of immunomodulators and overcomes HLA DQA1*05 effect.

## Introduction

Infliximab and adalimumab represent effective therapeutic options for the management of Inflammatory Bowel Disease (IBD) patients. However, approximately 30%-50% of the patients have a primary nonresponse (PNR) or secondary loss of response (SLR) to antitumor necrosis factor.^1,2^ PNR and SLR are often associated with suboptimals or undetectable drug levels, with or without the presence of anti-drug antibodies (ADAs).^3–6^

A follow-up analysis of the PANTS study, a prospective cohort designed to identify predictors of anti-TNF therapy failure, carrying HLA DQA1*05 gene variant—present in approximately 40% of European population—are a significantly increased risk of developing immunogenicity to anti-TNF agents.^7,8^

Immunogenicity to anti-TNF therapy can be reduced by using combination treatment with an immunomodulator, particularly in the case of Infliximab.^9–11^ Another recommended strategy to prevent anti-TNF failure is therapeutic drug monitoring (TDM).

Therapeutic drug monitoring has traditionally been used re-actively to guide drug intensification after a nonresponse to anti-TNF therapy.^12^ Proactive TDM (PTDM), which systematically measures drug serum levels during induction or maintenance to optimize concentrations, has gained attention for its potential to prevent SLR and improve outcomes, particularly in anti-TNF monotherapy.^13,14^

The TAXIT and TAILORIX trials, comparing PTDM to empirical dosing strategies, found no significant differences in clinical outcomes but noted a trend toward better pharmacokinetic profiles in the PTDM groups.^15,16^ The PAILOT study in pediatric patients observed significant improvements in clinical remission (CR) rates and normalization of CRP levels with PTDM.^17^ Retrospective cohorts suggest PTDM may reduce the association between the HLA DQA1*05 variant and SLR,^18,19^ though these studies used lower serum targets derived from earlier trials and lacked Bayesian pharmacokinetic modeling.

Dashboards-guided models predict optimal dosing intervals based on measured serum levels and individual clearance factors. The PRECISION trial demonstrated higher rates of sustained CR with dashboard-driven infliximab dosing over 1 year.^20,21^ Other prospective cohorts also reported improved clinical and pharmacokinetic outcomes using this approach for induction and maintenance, though serum targets for infliximab induction were also considered low (<10 µg/mL).^22,23^ In a post hoc analysis of the pediatric prospective cohort of PRECISION trial, the risk of immunogenicity was not associated with HLADQA1*05 variant.^24^

Challenges remain in determining optimal anti-TNF serum concentrations for varying clinical scenario, such as induction, histological healing, or penetrating CD, as well as standardizing drug level and ADAs measurement methods.^25^

The ongoing OPTIMIZE trial is evaluating PTDM guided by Bayesian dashboards versus standard of care in active CD, with higher infliximab serum targets (>17 μg/mL for induction, >10 μg/mL at week 14 and >7 μg/mL for maintenance).^26^

This study aims to provide real-world data on clinical, pharmacokinetic, and durability outcomes of Bayesian-guided PTDM for anti-TNF induction. Additionally, it seeks to assess the impact of this strategy on treatment efficacy and immunogenicity.

## Methods

### Population

We conducted a single-center, cohort study, involving patients aged over 18 diagnosed with CD and Ulcerative colitis (UC) who initiated treatment with adalimumab or infliximab, under a prospectively personalized pharmacokinetic-guided induction protocol, from January 2020 to March 2023. To be included in the analysis, patients were required to have received their first 3 induction doses, and drug serum levels had to be determined at least at weeks 2 and 6. Patients included were followed for at least 52 weeks or until treatment discontinuation.

### Description of the Pharmacokinetic Bayesian Model

The pharmacokinetic approach utilized pre established dashboard models based on Bayesian inference, conducted by the Pharmacy department. The adalimumab dashboard-guided model incorporated drug serum levels, body max index (BMI) and calprotectin.^27^ The infliximab model included drug serum levels, weight, albumin levels, the presence of ADAs, and concurrent use of immunomodulators.^28^ The proposed targeted serum levels at induction were >18 µg/mL for infliximab and 10-15 µg/mL for adalimumab. For maintenance the proposed levels were 5-8 μg/mL and 8-12 μg/mL, respectively.^29^

### Pharmacokinetic Protocol

We established a pharmacokinetic protocol for the Bayesian model approach, in collaboration with the Pharmacy and Immunology departments. Patients initiating adalimumab treatment received standard doses of 160 mg at week 0 and 80 mg at week 2. Serum drug levels were measured at weeks 2, 6, and 14, along with assessments of weight, albumin, CRP, and calprotectin. These parameters were inputted into the model to predict the optimal dose required to achieve the targeted levels. A similar protocol was applied to infliximab induction. Patients received standard doses of 5 mg/kg or 10 mg/kg, based on clinician criteria, at week 0 and 2. Serum drug levels were measured at weeks 2, 6, and 14, along with the aforementioned parameters, and inputted into the model to predict the optimal subsequent infliximab dose.

### Laboratory Tests

Drug serum concentrations and ADA were quantified using a chemiluminescent immuno-assay (CLIA) laboratory technique. The assay's determination range was from 0.3 to 24 μg/mL for adalimumab and infliximab serum concentrations. ADA presence was only assessed if adalimumab serum levels were <5 µg/mL or infliximab levels <3 μg/mL. The range of determination of ADA presence was from 10 to 2000 μg/mL.

### Data Collection

We retrospectively recorded data from electronic medical charts on demographic and baseline characteristics, including age, sex, IBD type, disease duration, previous biologic treatment, disease location, presence of perianal disease, use of steroids, or immunomodulator treatment during induction, type of anti-TNF therapy, HLA DQA1*05 status, and disease activity at week 0. Disease activity was assessed using the Simple Clinical Colitis Activity Index (SCCAI) for UC and the Harvey-Bradshaw index (HBI) for CD. Endoscopic activity was evaluated using the Simple Endoscopic Score for Crohn’s Disease (SES-CD) and the Mayo Index for UC.

### Outcomes Measure

Our primary outcome was anti-TNF durability, defined by the proportion of patients still receiving treatment at weeks 24 and 52. Secondary outcomes included the rate of CR at weeks 24 and 52, defined by an SCCAI < 2 or HBI < 5; endoscopic remission (ER) at week 52, defined by a SES-CD < 1 or a Mayo Index equal to 0 or 1; proportion of patients with an intensified dose at weeks 24 and 52, defined as any increase of drug dose above the standard (40 mg every 2 weeks for adalimumab or 5 mg/kg every 8 weeks for infliximab). We also investigated the influence of immunomodulator use, prior biologic treatments, and HLADQA1*05 status on the durability of the anti-TNF therapy.

### Statistical Analysis

Statistical analyses were performed using STATA (StataCorp. 2021. Stata Statistical Software: Release 17. College Station, TX: StataCorp LLC). Descriptive statistics were represented as median or mean for continuous variables and as numbers and percentages for qualitative variables. Survival analyses were estimated using Kaplan–Meier curves and compared using the log-rank test. Univariate and multivariate analysis were conducted. Categorical variables were compared using *χ*^2^ test or Fisher’s exact test, as appropriate, and continuous variables were compared using the Mann–Whitney *U*-test. Cox proportional hazards models were applied to explore the associations between drug survival and multiple variables. All independent variables with a potential association with drug survival in the univariate model were included in the multivariate model, along with those relevant to the study's objectives. The examined variables were CRP, albumin, calprotectin, sex, smoking habit, disease location, perianal disease presence, HLDAQA1*05 status, immunomodulator use, steroids use, prior-biological treatment, and drug serum levels of anti-TNF at weeks 2 and 6. A *P*-value < .05 was considered statistically significant.

## Results

### Patient Population

A total of 155 patients initiated anti-TNF treatment between January 2020 and March 2023. Eight patients were excluded: 3 did not complete drug induction, 3 had a supra-accelerated dosage at induction due to clinical severity, trough levels were not measured in 1 patient, and 1 patient was diagnosed of perianal disease without a diagnosis of IBD. Therefore, a total of 147 patients were included in the study, 110 with adalimumab treatment (74 CD and 36 UC) and 37 with infliximab (18 CD and 19 UC). Baseline characteristics of the population are shown in Table 1 and Supplementary Material 1.

**Table 1.** Baseline demographics.

Type of disease	
Crohn’s disease, *n* (%)	92 (62.5)
L1 L2 L3 L4	50 (53.7) 9 (9.6) 33 (35.4) 1 (1.1)
Ulcerative colitis, *n* (%)	55 (37.)
E1 E2 E3	2 (3.7) 23 (42.5) 29 (53.7)
Type of anti-TNF:	
Infliximab, *n*, (%)	37 (25)
Adalimumab, *n*, (%)	110 (75)
Disease duration (years), median (IQR)	7.3 (5.9-8.7)
Female, *n* (%)	75 (51)
Age, median (IQR)	44 (41.5-46.4)
nonsmoker, *n* (%)	78 (48.3)
Smoker, *n* (%)	35 (23.8)
Former smoker, *n* (%)	41 (27.9)
BMI, median (IQR)	24.5 (23.6-25.5)
HLADQ A1*05 carriers, *n* (%)	64 (44.1)
Perianal fistulizing disease, *n* (%)	22 (14.9)
CRP (g/dL), median (IQR)^a^	1.5 (1.1-2.0)
Albumin (g/dL), median (IQR)^b^	4.0 (4.0-4.1)
Calprotectin (mg/kg), median (IQR)^c^	1033.4 (70.0-1996.0)
Immunomodulator^d^ combined, *n* (%)	67 (45.5)
Previous biologics, *n* (%)	17 (11.5)
Infliximab, *n* (%) Adalimumab, *n* (%) Vedolizumab, *n* (%) Ustekinumab, *n* (%)	4 (2.7) 3 (2.1) 5 (3.4) 5 (3.4)
Steroids at inclusion, *n* (%)	66 (44.9)
Clinical remission at inclusion, *n* (%)	88 (59.8)
Perianal disease, *n* (%)	22 (14.9)
Endoscopic remission at inclusion, *n* (%)	8 (5.4)

Abbreviations: *n*, number of subjects; IQR, interquartile range.

^a^Data of CRP were missing for 1 patient.

^b^Data of albumin were missing for 1 patient.

^c^Data of calprotectin were missing for 77 patients.

^d^All patients with immunomodulator were on thiopurine.

### Drug Persistence

Five patients (3.4%) ceased treatment before reaching week 24 due to lack of efficacy. By week 52, the number of patients discontinuing treatment had risen to 37 (25.1%). Among these, 28 discontinued due to lack of efficacy, 7 due to severe psoriasis development, 1 due to surgical indication, and 1 due to immunogenicity. Median follow-up duration was 22.4 months (IQR 20.6-24.3), 11.6 months (IQR 8.6-14.2) in patients who stopped the treatment compared to 26.1 months (IQR 24.2-27.9) in those who continued it.

**Figure 1.**
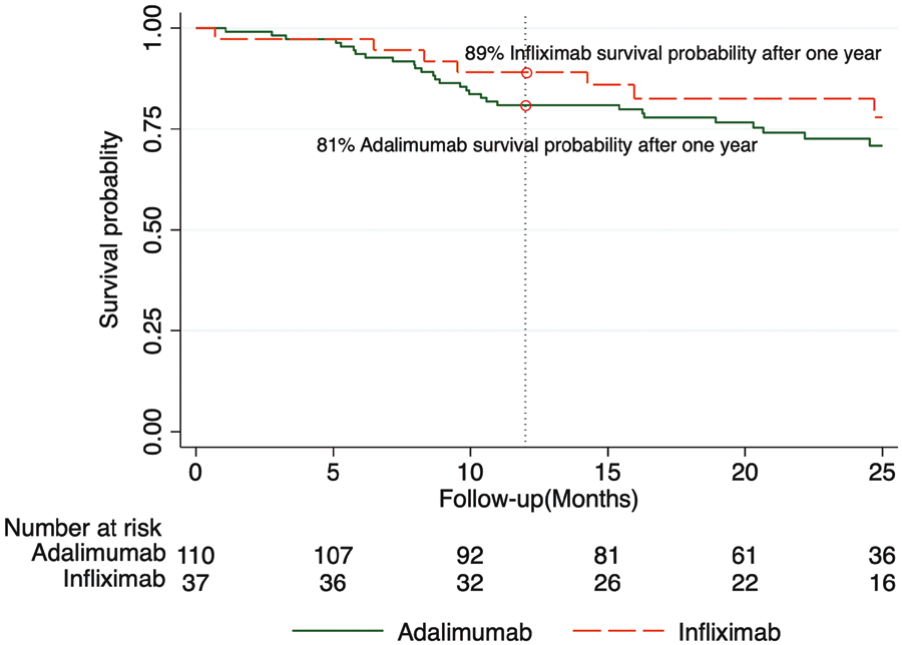
Drug survival probability by anti-TNF drug. Log-rank test *P* = 0.45

**Figure 2.**
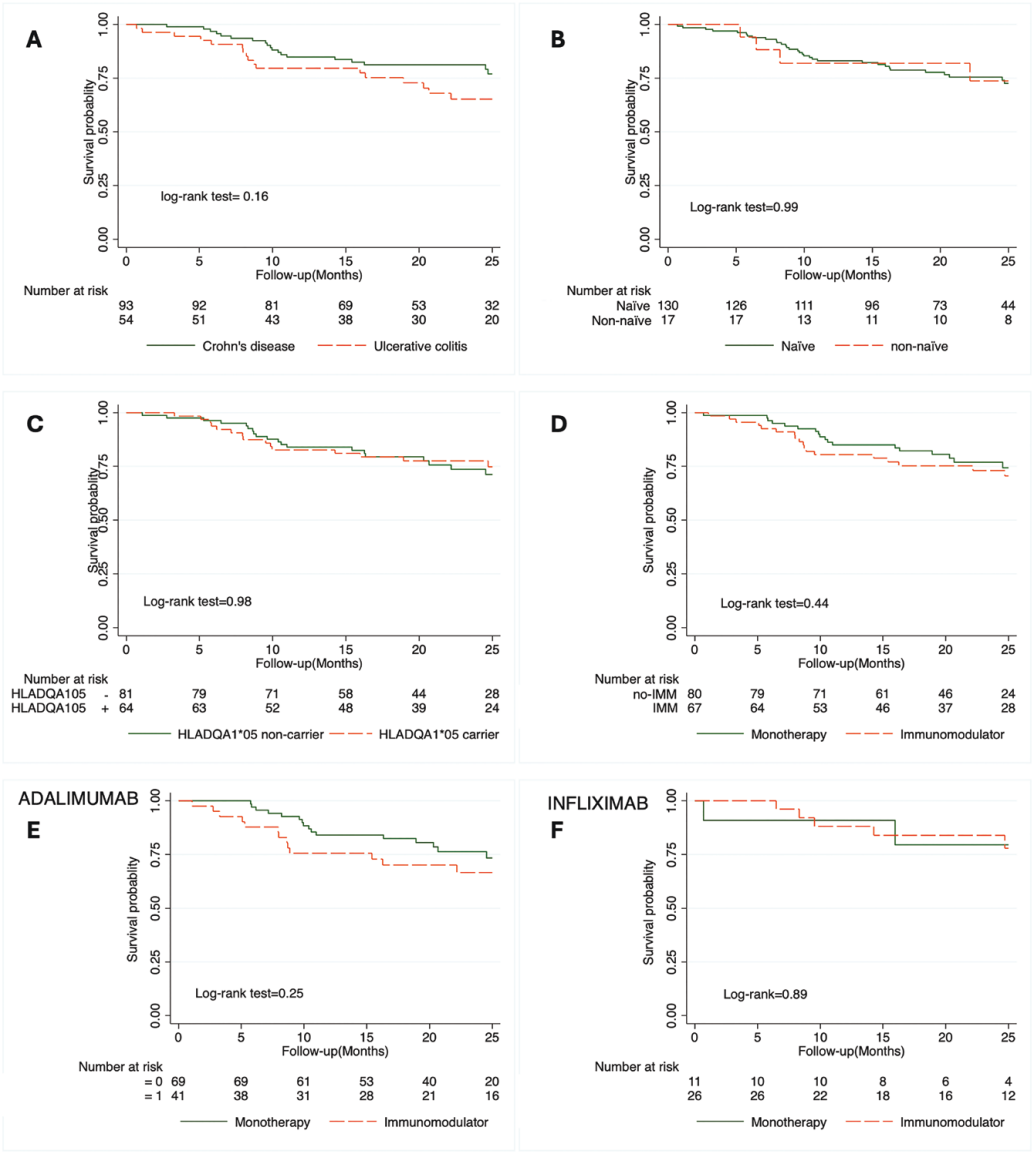
Drug survival probability after subgroup analysis by potentially immunogenic covariates. A, By type of disease; B, by previous biologic use; C, by HLA DQA1*05 status; D, by immunomodulator use; E, adalimumab by immunomodulator use; F, Infliximab by immunomodulator use. Abbreviations: CD, Crohn’s disease; IMM, immunomodulator; UC, ulcerative colitis.

The overall probability of anti-TNF drug survival after 1 year was 85.0%. Although patients undergoing infliximab treatment showed numerically higher treatment survival rates compared to those on adalimumab, this difference did not reach statistical significance (Figure 1). Additionally, no discernible differences in drug survival probability were observed when stratified by type of anti-TNF, type of IBD, use of immunomodulator, HLA DQA1*05 status, or prior biologic use, as depicted in Figure 2.

### Dashboard Model and Pharmacokinetic Outcomes

**Figure 3.**
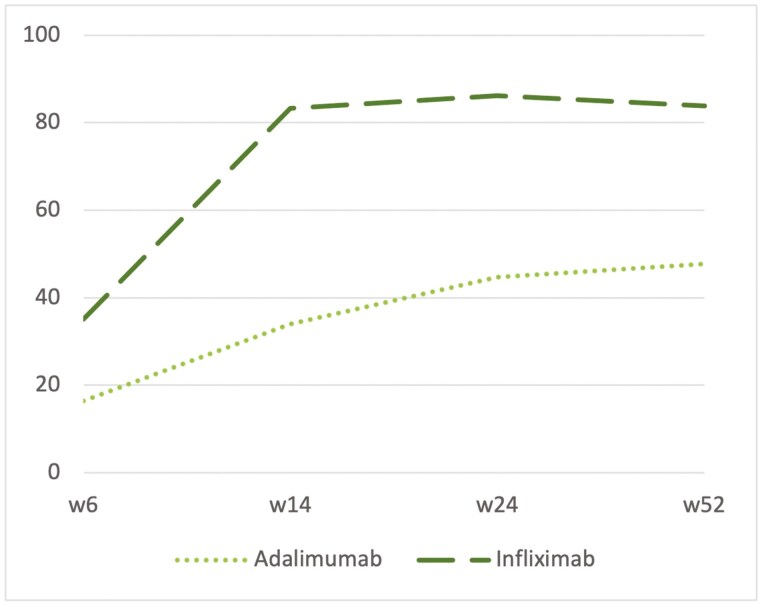
Percentage of dose intensification during follow-up. Abbreviation: w, week.

The proportion of patients receiving intensified doses increased during follow-up after implementing the dashboard model, with dose intensification beginning at the third infusion for both treatments (Figure 3). By week 6, 21.1%. (31/147) of patients had undergone dose intensification based on week 2 levels. By week 14, 46.2% (67/147) had dose intensification, guided by week 6 levels as well as week 2 levels, since the model incorporates previous levels and parameters to make predictions. Specifically, after 52 weeks, 58.6 % (71/121) of patients were prescribed an intensified dose, with 77.7% (28/36) in the infliximab group and 39.0% (43/110) in the adalimumab group.

We did not observe significant differences in anti-TNF drug serum levels among different types of diseases, nor did we find any influence of HLADQA1*05 variant presence, use of immunomodulator or previous biologic use. Notably, only 1 patient treated with adalimumab developed ADAs (Supplementary Material 2).

### Clinical Outcomes

By week 24, 92.5% (*n* = 136) out of the 147 enrolled patients were in CR. Among them, 142 out of 147 completed the initial 24-weeks follow-up period. The rates of CR at week 24 were comparable between the adalimumab and infliximab groups, standing at 92.7% (102/110) and 91.9% (34/37), respectively. By week 52, 72.7% (107/147) of the patients remained in CR, with 68.1% (75/110) in the adalimumab group and 86.4% (32/37) in the infliximab group. At this time, only 2 patients in the adalimumab group were receiving steroid treatment, one in CR, while none in the infliximab group (Supplementary Material 3).

### Endoscopic Outcomes

Ileocolonoscopy was conducted in 133 out of 147 patients between weeks 24 and 52. However, ileoscopy could not be complete in 15.8% (21/133) of these patients due to stricturing CD. At the end of the follow-up period, ER was achieved in 53.7% (79/147) of the patients, with 64.8 % (24/37) in the infliximab group, and 39.0% (43/110) in the adalimumab group. One patient in the adalimumab group who was in ER was receiving steroid treatment (Supplementary Material 4).

### Factors Associated with Clinical Outcomes and Drug Durability

In our multivariate analysis, we found that patients who were naïve to biologic therapy demonstrated higher rates of CR at week 24 (*P* = .006). Additionally, the prescription of an intensified dose by week 52 was associated with increased rates of ER (*P* = .013). Interestingly, according to Cox proportional hazard models, only the prescription of an intensified dose by week 52 was linked to improved drug survival (Table 2).

**Table 2.** Multivariate associations with clinical remission (CR), ER, and drug survival.

Variable	Drug survival			Clinical remission			Endoscopic remission		
	HR	95% CI	*P*	OR	95% CI	*P*	OR	95% CI	*P*
Type of anti-TNF	**0.57**	0.1-2	*.38*	**5.3**	0.006-2.9	*.2*	**2.5**	0.8-7.3	*.98*
HLA DQA1*05 carrier	**0.85**	0.2-2.7	*.79*	**0.25**	0.01-6.3	*.2*	**1**	0.44-2.4	*.08*
Immunomodulator	**1.5**	0.4-4.7	*.45*	**0.13**	0.005-3.2	*.21*	**1.13**	0.47-2.6	*.7*
Naïve to biologic	**1.2**	0.2-5.8	*.27*	**9.8**	3.7-26	***.006***	**0.45**	0.12-0-7	*.24*
Intensified dose prescribed at w52	**5.1**	1.1-24	***.039***	**0.52**	0.019—14	*.7*	**0.3**	0.01-0.5	***.008***

Abbreviations: anti-TNF, antitumor necrosis factor; CI, confidence interval; HR, hazard ratio; OR, odds ratio; *P*, *P*-value; w52, week 52. HR and OR values are highlighted in bold; statistically significant values (*P* <.05) are shown in bold italics.

Moreover, factors such as CRP, albumin, calprotectin, the type of anti-TNF, age, sex, smoking habit, disease duration, HLA DQA1*05 carriers, or the use of concomitant immunomodulator did not exert any significant impact on clinical or pharmacokinetic outcomes.

## Discussion

In our study, involving 147 patients undergoing anti-TNF induction with model-dosing adjustment based on a Bayesian system, the probability of anti-TNF drug survival reached 85.0% after 1 year, with only one out of 147 patients developing antibodies to adalimumab. Furthermore, a substantial proportion of patients achieved clinical and ER at weeks 24 and 52. Multivariate analysis revealed no factors influencing the primary endpoints, indicating no discernible differences between HLA DQA1*05 carriers and non carriers as well as no differences between patients with or without concomitant immunomodulators use.

These real-life findings parallel those of the PRECISION trial, endorsing dashboard modeling to optimize ant-TNF treatment maintenance.^20^ However, it's worth noting that patients in the PRECISION trial, initiated treatment while in CR, leading to a better clinical outcome and a more favorable pharmacokinetic profile, compared to those with active disease or a higher inflammatory burden. Half of our patients had clinically active disease before starting anti-TNF induction, with only 8 out of 147 in ER before inclusion. Despite this challenging patient situation, the use of a PTDM strategy guided by a Bayesian model resulted in 70.0% of patients in CR and almost 60.0% of them also attaining ER after 1 year. Our findings also corroborate previous studies highlighting the advantages of PTDM in averting SLR. This is likely attributed to the correlation previously demonstrated by the PANTS cohort study between low serum levels of anti-TNF at induction and the development of ADAs, resulting in reduced exposure to the drug.^7^

Dashboard modeling effectively maintains optimal drug exposure by predicting the necessary doses required to achieve target levels in specific patients. This approach helps to prevent immunogenicity and enhances the likelihood of anti-TNF survival. Our findings support this notion, as evidenced by the low rates of immunogenicity observed in our cohort, only 1 patient developed ADAs, and the high probability of drug survival after 1 year (85.0%).

However, over half of our patients required an intensified dose during follow-up to achieve the higher proposed target drug levels. Previous studies suggested lower target levels, primarily based on pilot trials of anti-TNF therapies. Therefore, achieving the higher proposed target drug levels requires more frequent intensified dosing. The first study to consider higher Infliximab levels at maintenance, >10 μg/mL, was the PTDM study conducted by Dubinsky et al. In their study, most of the patients required intensified dosing by week 14, consistent with our findings. However, it is important to note that the population of their study primarily consisted of pediatric patients, who are known to have a higher rate of drug clearance compared to adults.^21^

Another benefit of the dashboard-guided dosing strategy is its ability to obviate the need for concomitant immunomodulators, thus favoring the pharmacokinetic profile of the anti-TNF drug. Traditionally, combination therapy with anti-TNF, particularly with infliximab, has been preferred to mitigate immunogenicity and enhance drug durability. However, as previous studies have shown, when employing a PTDM strategy, no significant differences were observed compared to monotherapy treatment.^14^ The potential advantage of monotherapy treatment is the reduction of adverse events associated with combination therapy, such as risk of infections or malignancies.

Several factors are known to be associated with an increased risk of immunogenicity, including young age, BMI, smoking habit, high inflammatory burden, drug holidays, or poor adherence. Interestingly, none of these factors were found to be associated with immunogenicity in our population, suggesting that the Bayesian model can mitigate the effects of these factors on drug clearance.

HLA DQA1*05 carriers, which constitute approximately 50.0% of the European population, have been identified in the PANTS cohort as having a higher risk of immunogenicity. However, our findings, along with data from a post hoc analysis of the PRECISION trial, suggest that this immunogenicity appears to be mitigated after implementing a dashboard-guided dosing model.^24^ Consequently, we found a similar survival probability of the drug after 1 year, independently of the presence of known pro-immunogenic factors, as depicted in Figure 2.

To our knowledge, this is the first study assessing the utility of the dashboard model in anti-TNF induction, incorporating protocolized pharmacokinetic monitoring at weeks 2, 6 and 14. We have the advantage of complete 1-year follow-up data for all patients, encompassing all clinical outcomes and nearly all patients with endoscopic assessment. Additionally, we meticulously tracked the pharmacokinetic profile by measuring drug levels and ADA for all included patients at various time-points.

Our study has several limitations. First, the data were recorded retrospectively from electronic medical charts, leading to some missing variables. This may partly explain the observed discordance between clinical and ER prior to initiating treatment. All patients were included in the final analysis without assessing the nature of the treatment failure, which may have led to a misinterpretation of the true CR in a homogeneous group of patients. Moreover, it is a single-center cohort comprising a heterogenous group with both CD and UC patients initiating infliximab and adalimumab. Due to the standardized protocol of dashboard model-guided induction for all anti-TNF patients, a comparison group was not included. We did not use a historical comparison group due to the variability in managing anti-TNF serum levels in the years preceding the implementation of the dashboard model.

Nevertheless, limitations persist regarding the PTDM dashboard strategy. Firstly, determining the optimal drug level threshold to target poses a challenge, as therapeutic thresholds appear to vary depending on factors such as the phenotype, inflammatory burden, or treatment goals. Our target thresholds, as previously described, are based on published literature, and applied universally to all patients, except for cases involving perianal disease, where higher drug serum levels are needed. However, achieving more ambitious goals, such as endoscopic or histological remission, may necessitate even higher target levels.^30^

Another limitation of PTDM concerns the variability in assays used to assess drug concentrations or ADAs, resulting in discrepancies between studies. While our practice relies on the CLIA technique, known for its availability and rapid results, most studies employ the enzyme-linked immunosorbent assay (ELISA). Moreover, our hospital lacks ADA drug-sensitive assays, potentially leading to an underestimation of ADAs formation.

Additionally, the dashboard model does not consider clinical or endoscopy activity; dose intensification is only based on drug serum levels. Consequently, these patients may have been in clinical or endoscopy remission without necessitating dose intensification. Furthermore, the retrospective design meant that radiologic tests were not standardized during follow-up, resulting in an inability to assess inflammatory status for patients who did not undergo complete ileocolonoscopy due to luminal stenosis. Such cases were classified as having an SES-CD greater than 2, indicative of active disease. Additionally transmural remission through radiological methods has not been assessed.

In summary, our study provides compelling data on the effectiveness of a pharmacokinetic dashboard strategy during anti-TNF induction, effectively mitigating immunogenicity and anti-TNF failure, even without the use of immunomodulators or in HLA DQ1*05 carriers. We believe this supports the implementation of pharmacokinetic management of anti-TNF therapy in IBD centers to optimize patient outcomes. Prospective, well-designed studies are required to demonstrate the superiority of the pharmacokinetic approach to anti-TNF induction.

## Supplementary Material

otaf023_suppl_Supplementary_Materials

## Data Availability

The data supporting the findings of this study can be made available from the corresponding author upon reasonable request.
