# Supplementary material for: Dashboard-Guided Anti-TNF Induction: An Effective Strategy to Minimize Immunogenicity While Avoiding Immunomodulators—A Single-Center Cohort Study
Source: Crohns Colitis 360. 2025 Jun 27;7(3):otaf023. doi: 10.1093/crocol/otaf023 (PMC12260160; doi:10.1093/crocol/otaf023)

**Supplementary material 1: Baseline demographics stratified by disease and treatment**

| Characteristics | Infliximab and CD | Infliximab and UC | Adalimumab and CD | Adalimumab and UC |
| --- | --- | --- | --- | --- |
| Crohn’s disease, n (%) | 18 |  | 74 |  |
| L1  L2  L3  L4 | 7 (36.8)  4 (21.0)  8 (42.1) |  | 43 (58.1)  5 (6.7)  25 (33.7)  1 (1.3) |  |
| Ulcerative colitis, n (%) |  | 19 |  | 36 |
| E1  E2  E3 |  | 7 (38.9)  11 (61.1) |  | 2 (5.5)  16 (44.4)  18 (50.0) |
| Disease duration (years), median (IQR) | 8.05 (4.7-11.3) | 6.9 (3.5-10-3) | 6.6 (4.4-8.7) | 8.7 (5.7-11.6) |
| Female, n (%) | 14 (73) | 8 (44) | 38 (52) | 16 (44) |
| Age, median (IQR) | 41.3 (36.05-46.5) | 46.9 (38.5-55.36) | 43.4 (39.6-47.27) | 44.6 (39.7-49.5) |
| Non-smoker, n (%) | 8 (42) | 9 (50) | 40 (54) | 8(22) |
| Smoker, n (%) | 7 (37) | 2 (11) | 17 (23) | 14(39) |
| Former-smoker, n (%) | 4 (21) | 6 (39) | 15 (21) | 14(39) |
| BMI, median (IQR) | 26.9 (22.9-30.8) | 24.7 (21.4-28.1) | 23,7 (22.4-24.9) | 25 (23.3-26.7) |
| HLA DQA1*05 carriers, n (%) | 7 (38.9) | 11 (56.2) | 33 (44.5) | 14 (38.8) |
| Immunomodulator combined, n (%) | 10 (55.5) | 16 (84.2) | 21 (2) | 19 (52.7) |
| Previous biologics, n (%) | 5 (27.1) | 2 (10.5) | 5 (6.7) | 5 (13.8) |
| Infliximab, n  Adalimumab, n  Vedolizumab, n  Ustekinumab, n | 3  2 | 2 | 1  1  3 | 3  2 |
| Steroids at inclusion, n (%) | 8 (47.3) | 14 (72.2) | 22 (29.7) | 19 (52.7) |
| Clinical remission at inclusion, n (%) | 13 (68.4) | 3 (16.6) | 57 (77.0) | 15 (41.6) |
| Perianal disease, n (%) | 7 (36.8) | 1(5.5) | 10 (13.5) | 3 (8.3) |
| Endoscopic remission at inclusion, n (%) | 0 (0) | 0 (0) | 3 (4.1) | 5 (13.8) |

*Abbreviations: CD: Crohn’s disease; UC: Ulcerative colitis; BMI: body mass index; IQR: interquartile range*

**Supplementary material 2: Medium anti-TNF drug serum levels, after subgroup analysis by disease type and type of during follow up**. ADL: Adalimumab; IFX: Infliximab

**
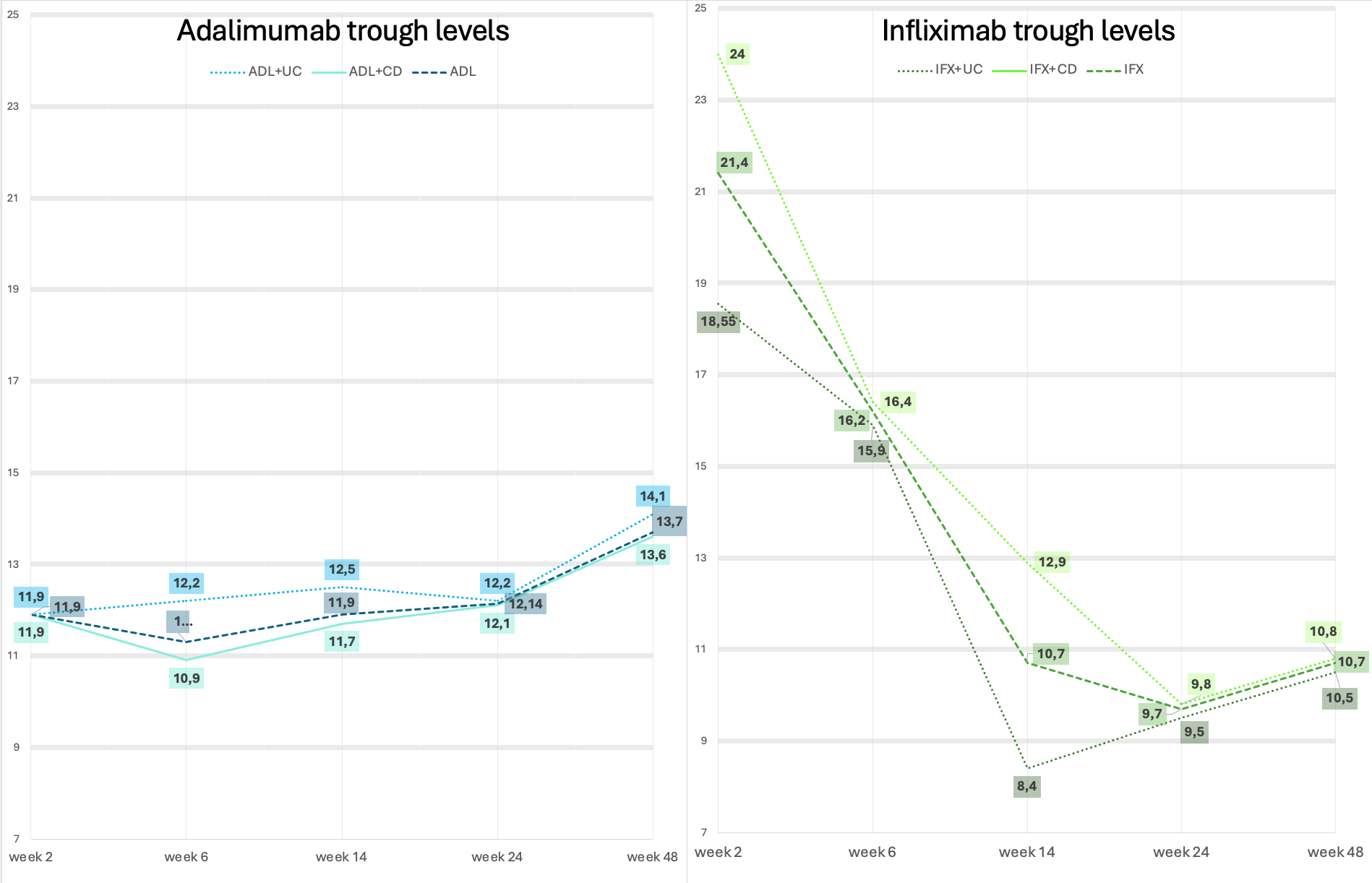
**

**Suplementary material 3. Clinical remission rates (%) during the follow-up after subgroup analysis by disease type and drug.** Abbreviations: ADL, Adalimumab; UC, Ulcerative colitis; CD, Crohn’s disease; IFX, Infliximab; CR, Clinical remission; w, week


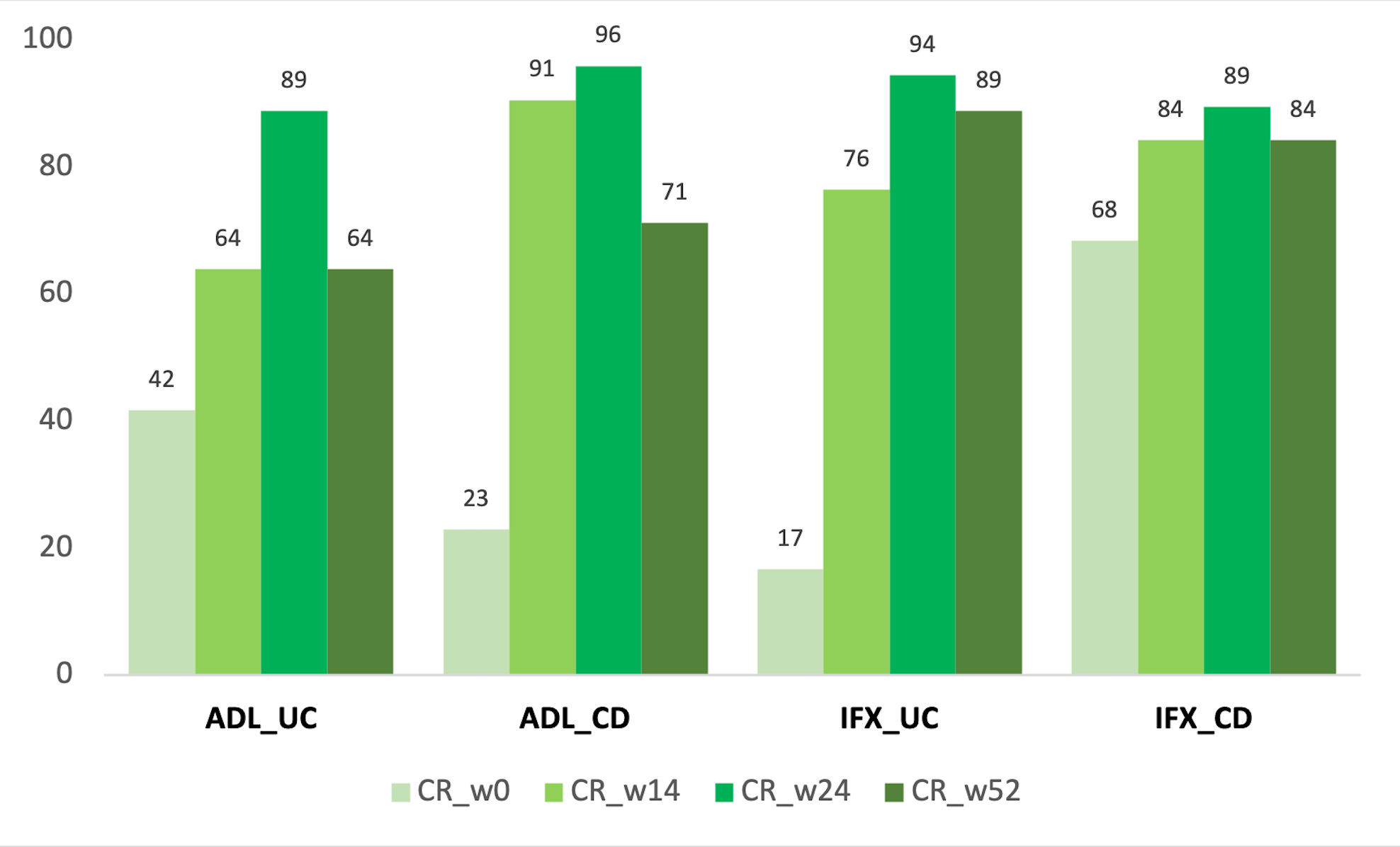


**Supplementary material 4: Endoscopic remission rates (%) after week 24, following subgroup analysis by. disease type and drug.** Abbreviations: ADL, Adalimumab; UC, Ulcerative colitis; CD, Crohn’s disease; IFX, Infliximab; ER, Endoscopic remission


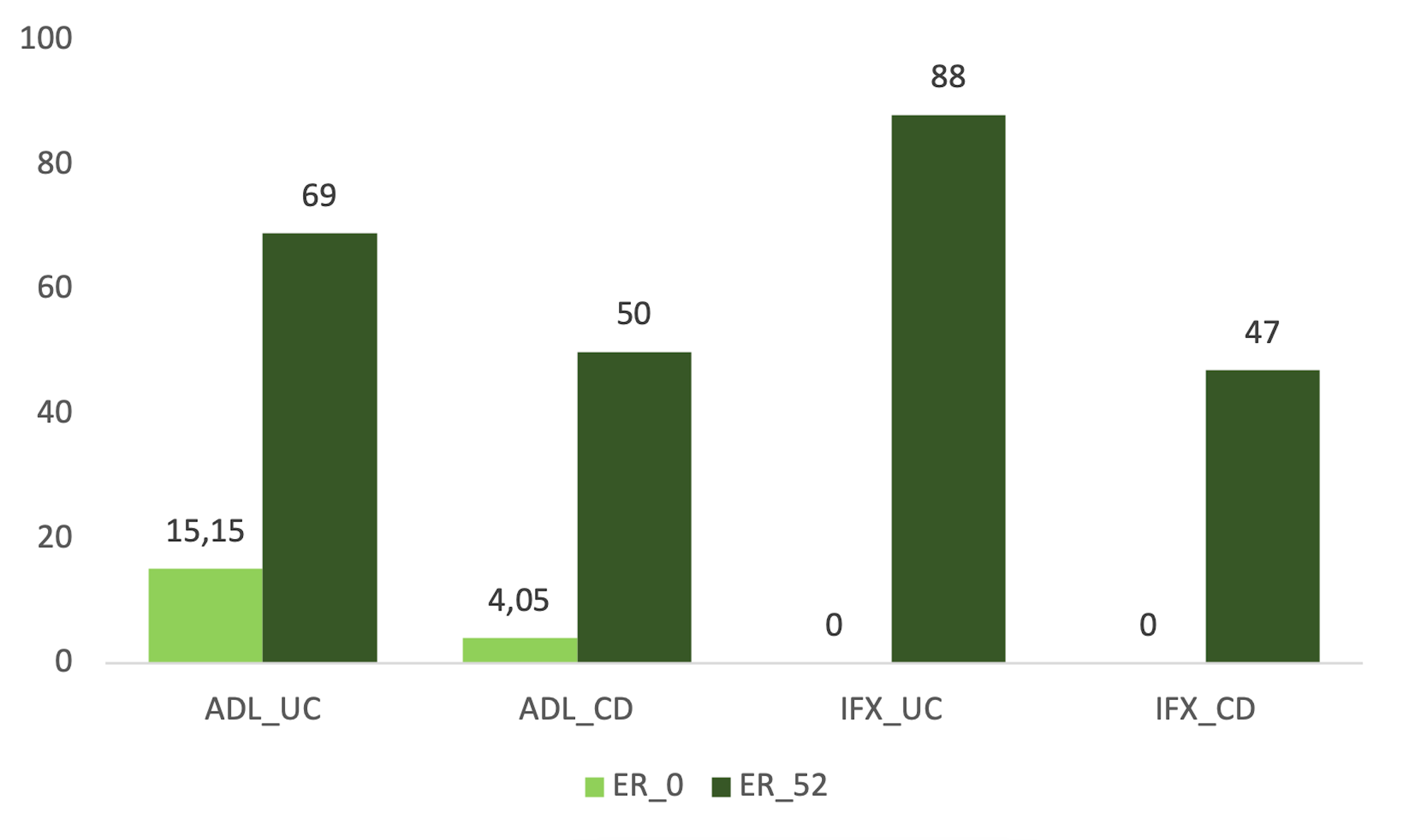

Supplement: otaf023_suppl_Supplementary_Materials [file otaf023_suppl_supplementary_materials.docx]
